# Marijuana use and high-risk health behaviors among diverse college students post- legalization of recreational marijuana use

**DOI:** 10.1016/j.puhip.2021.100195

**Published:** 2021-10-02

**Authors:** Laura Chandler, Aimn W. Abdujawad, Sinjini Mitra, Archana J. McEligot

**Affiliations:** a Department of Public Health. California State University, Fullerton, 800 N. State College Blvd., Room KHS-121, Fullerton, CA, 92834, USA; b ISDS Department of Information Systems and Decision Sciences, California State University, Fullerton, USA

**Keywords:** Marijuana, Health-risk behaviors, College students, Post legalization

## Abstract

**Objectives:**

This study examined high-risk health behaviors in marijuana-users among a diverse college population in Southern California, post legalization of marijuana for recreational use.

**Study design:**

A cross-sectional research design was employed utilizing existing data via the 2018 National College Health Assessment (NCHA) from a large Minority-Serving Institution (MSI) population [n = 1345 (Hispanic/Latino/a, n = 456; White, n = 353; Asian Pacific Islander (API), n = 288; Multiracial/Biracial, n = 195; Other, n = 53)].

**Methods:**

Chi square and *t*-tests assessed differences in descriptive characteristics (age, gender, race/ethnicity and GPA) and high-risk behaviors (alcohol, tobacco and sexual behaviors) among marijuana users and non-users. Logistic regression analyses examined the relationship between race/ethnicity and high-risk behaviors with marijuana use (dependent variable).

**Results:**

Among marijuana-users, significant (*p* = 0.004) differences were observed between race/ethnicity with Whites reporting using most (32.7%), followed by Hispanics (27.6%) and then APIs (17.8%). Marijuana-users compared with non-users consistently reported high-risk alcohol behaviors (*p* < 0.0001), were more likely to smoke tobacco (*p* < 0.0001) and engaged in more high-risk sexual behaviors (*p* < 0.0001). Logistic regression showed after adjusting for demographic characteristics and high-risk behaviors, race/ethnicity was borderline significantly associated with marijuana use, specifically for Whites (OR = 1.53; 95% CI: (−0.01, 0.86), *p* = 0.06) and the *Other* race/ethnicity category (OR = 2.32; 95% CI: (0.12, 1.56), *p* = 0.02) compared with APIs.

**Conclusion:**

Our findings clearly demonstrate deleterious high-risk behaviors such as alcohol use, tobacco use, and certain sexual behaviors occur more among marijuana-users compared to non-users, post legalization of marijuana for recreational use. Further, race-ethnic differences were observed. Therefore, continued examination of marijuana use trends and high-risk behaviors is critical in monitoring the implications of marijuana policy changes, specifically in diverse populations.

## 1. Introduction

As of April 2021, 36 states and the District of Columbia have passed laws that allow for the medical use of marijuana and 16 states and the District of Columbia have passed laws that legalize recreational use of marijuana; more states appear to be heading toward similar legislation [1]. Even though the debate on marijuana’s safety and benefits continues to be fiercely debated [2], public opinion on the legalization of marijuana has grown more favorable. In 1969 only 12% of the adult population supported legalization compared to 66% in 2019. Among the 18-34-year-old subgroup, support was even higher with 81% favoring legalization in 2019 [3]. With this data, it is not surprising that marijuana use has also gradually increased with college age adults, 18–25 years, experiencing the greatest growth from 17.3% in 2002 to 22.1% in 2018 [4]. Furthermore, in their examination of national survey results on drug use from 1975 to 2016, Schulenberg and colleagues [5] noted historical trends of marijuana use increased at different levels and for different lengths of time across younger ages, 19–20 years through 29–30 years, with almost all age groups reporting increasing prevalence from 2010 onward.

Marijuana use has been shown to have a negative impact on the lives of college students [6-9]. Earlier studies have shown numerous negative associations with marijuana use and academic success indicators such as lower GPA, reduced studying time, late assignments [6,8], missing class and discontinuous enrollment in college [7,9]. Furthermore, the heavier the marijuana use, the more likely negative outcomes were reported [10]. Importantly, the majority of the studies investigating negative impacts and marijuana use in college students are prior to legalization of recreational marijuana [6-9], and even less of this data are available on a diverse student population.

Adverse high-risk behaviors, including tobacco use, binge drinking and use of other illicit drugs have also been associated with marijuana use in college students [11,12]. Using marijuana and tobacco at the same time may lead to increased exposure to harmful chemicals, causing greater risks to the lungs, and the cardiovascular system [13]. According to the National Survey on Drug Use and Health, alcohol and marijuana were substances most frequently used by college students (18–22 years old) [4,14]. In addition, among users of both substances, alcohol and marijuana have been shown to be more concurrently used rather than alone [15].

Another area of concern is sexual risk behaviors. Marijuana use, among adolescents and young adults, has been associated with sexual risk behaviors and outcomes, including inconsistent condom use, multiple sexual partners, and STI diagnoses [16-18]. Bryan and colleagues [17] showed that marijuana use increased the likelihood of intercourse due to reduced inhibitions and effects on cognitive ability. In addition, they noted a decreasing ability to negotiate and carry out condom use. Furthermore, Metrik and colleagues [19] found that both alcohol and marijuana use were independently correlated with greater odds of having casual sexual intercourse.

Data from the National Survey on Drug Use and Health also showed that among 18-24-year-old respondents, Blacks and Hispanics experienced more marijuana use disorder than other ethnic groups [13]. Keyes and her colleagues [20] found similar results when examining race/ethnicity in Monitoring the Future data from 2006 to 2015, pre-legalization. However, they note the importance of a deeper look into diverse populations as they also found confounding factors of class size for Black high school seniors and urban setting for Hispanic seniors [20].

With legalization legislation in the United States beginning only a decade ago and in California as recently as 2016, there is limited data available on high-risk health behaviors and marijuana use postlegalization in college-aged students. Even more limited are those that focus on ethnically diverse college groups [21-23]. Therefore, this study examined health behaviors in marijuana-users post legalization in a diverse, urban college population in Southern California. Specifically, we investigated alcohol and tobacco use, as well as sexual behaviors among marijuana-users compared with non-users post legalization in a racially/ethnically diverse college group. We hypothesize that marijuana-users will partake in increased high-risk behaviors compared with non-users, and that use will vary by race/ethnicity.

## 2. Methods

The National College Health Assessment (NCHA) is a nationally recognized research survey designed to collect data on students’ health habits, behaviors, and perceptions. NCHA data collected from a large Minority-Serving Institution (MSI) in Southern California (approximately 40,000 students), during the spring semester of 2018 was utilized for the present study. The assessment consists of 60 questions and approximately 300 items addressing a range of student health topics such as alcohol, tobacco and other drug use; sexual health; nutrition and exercise; mental health; and personal safety and violence. For the present study, a computer-generated random sample of 10,000 students were invited, via email, to complete the online survey from March 5, 2018 to March 23, 2018 with periodic reminders being sent to those yet to complete it. The response rate was 14.28% equating to n = 1428 participants completing the survey. Of the 1428 respondents, 1345 had complete data on demographics (Hispanic/Latino/a, n = 456; White, n = 353; Asian/Pacific Islander n = 288; Multiracial/Biracial, n = 195; Black, n = 32; Native American, n = 21), marijuana use, and other health-risk behaviors, and were included in the present study. Approval was secured by the campus’ Institutional Review Board, IRB #17-18-80.

## 3. Measures

### 3.1. Student demographics

Participants provided demographic information such as age, gender, and race/ethnicity. In addition, academic performance, via grade point average (GPA) was also ascertained. Race/ethnicity categories were as follows: White, Black, Hispanic or Latino/a (abbr. Hispanic for this study), Asian/Pacific Islander (API), American Indian, Alaskan Native, Multiracial/Biracial (abbr. Multiracial for this study), and Other.

### 3.2. Marijuana and tobacco use

Questions related to tobacco and marijuana use were asked as follows: *“Within the last 30 days, on how many days did you use the following…”* Response options included: Never used; Have used, but not in last 30 days; 1–2 days; 3–5 days; 6–9 days; 10–19 days; 20–29 days; and used daily.

### 3.3. Alcohol consumption

Three questions assessed alcohol consumption. Prior to answering questions related to alcohol, the instrument provided a clear definition/standardization pertaining to the term “drink” as follows: *One drink of alcohol is defined as a 12 oz. can or bottle of beer or wine cooler, a 4 oz. glass of wine, or a shot of liquor straight or in a mixed drink*. To assess alcohol consumption, participants were queried as follows, *the last time you “partied”/socialized, how many drinks of alcohol did you have?* A blank space was provided for subjects to type in a number; directions advised subjects to enter 0 (zero) if they did not drink alcohol. Additionally, to assess high-risk behaviors/binge drinking related to alcohol consumption, an item was included with two parts and queried participants to refer to the last 30 days when answering the following: *Did you drive after drinking any alcohol? and Did you drive after drinking five or more drinks of alcohol?* The options provided for both were: N/A don’t drive; N/A don’t drink; No; or Yes.

### 3.4. Sexual behavior and contraception

Questions related to sexual behavior were as follows: *Within the last* 12 months, *with how many partners have you had oral sex, vaginal intercourse, or anal intercourse?* A blank space was provided for subjects to type in a number; directions advised subjects to enter 0 (zero) if they did not have a sex partner within the last 12 months. Other questions were as follows: *Within the last 30 days, how often did you or your partner(s) use a condom or other protective barrier (e.g., male condom, female condom, dam, glove)* during vaginal sex? The response options included two nonapplicable selections reflective of one who had *never did this sexual activity or one who had not done this sexual activity during the last 30 days*. The other response options listed were: Never; Rarely; Sometimes; Most of the time; and Always.

## 4. Statistical analyses

The data were analyzed using both descriptive and inferential statistical methods. Descriptive statistics were calculated for the demographic data including age, gender, ethnicity, and GPA. Chi-square tests were conducted to study differences in gender, ethnicity and GPA between marijuana-users and non-users, and *t*-test assessed difference in age.

The response variable, marijuana use was dichotomized as follows: users were categorized as those indicating any use in the past 30 days and *non-users* or *never used* as those indicating no use within the past 30 days. For the race/ethnicity category, the variable was categorized as: White, Hispanic/Latino/a, Asian/Pacific Islander, Biracial/Multiracial and Other. Black, and American Indian/Alaskan Native were included in the “Other” category due to small sample sizes. All analyses were performed using SPSS version 25.

We initially conducted univariate analyses, *t*-test and chi-square test, respectively, to assess differences in various high-risk behaviors between marijuana-users and non-users. First, mean number of drinks consumed while socializing was calculated and compared between the two groups. Participants were also asked about alcohol consumption (binge drinking) during the past two weeks, and whether they consumed five or more drinks at a sitting. Responses were on an ordinal 8-point Likert scale with 1 = *N/A don’t drink* through to 8 = *6 times* (engaged in binge drinking). Mean differences in binge drinking were calculated on the ordinal scale (as a continuous variable) between marijuana-users and non-users. Also, we conducted chi-square analyses to assess differences in drinking after driving both for drinking any alcohol at all, as well as binge drinking (five or more drinks) between marijuana-users and non-users. Second, differences in tobacco use between the two groups were assessed. Tobacco use was categorized as *no* or *yes*, with *no* indicating never use or no use in the last 30 days and *yes* indicating any use of tobacco within the past 30 days. Last, we measured differences in sexual behaviors, including number of sexual partners (continuous) and condom use (for vaginal sex) in the last 30 days via *t*-test and chi-square, respectively, between marijuana-users and non-users.

Logistic regression was used to assess the association between race/ethnicity and high-risk behaviors with marijuana use compared to no use. The analysis population included only respondents with non-missing data on marijuana use, and race/ethnicity and high-risk behavior data. The dependent variable, marijuana use was dichotomized as use/no use, while independent variables included the following: Age (years, continuous), gender (male vs. females), race/ethnicity [White, Hispanic, Multiracial, Other, API (ref)], GPA (continuous), # of drinks at last party (continuous), # of sexual partners (continuous) and tobacco use (yes vs. no).

## 5. Results

Descriptive statistics on demographic data and GPA on the entire sample are presented in Table 1. Sample mean age was 23.10 years (±5.66 years) with majority being female (74.5%). Race/ethnicity was predominantly equally distributed between White (26.2%), Hispanic/Latino/a (33.9%) and API (21.4%) followed by Multiracial/Biracial (14.5%) and Other (4.0%).

Table 2 compares demographic and academic performance data between and within marijuana-users and non-users. Of the 1345 survey respondents, 275 (20.4%) were marijuana-users. Demographic data showed that marijuana-users were approximately one year younger (22.27 years) than non-users (23.19 years), indicating that younger college students were significantly more likely to use marijuana (*p* = 0.016). The proportion of users were more likely to be male, although not statistically significant. Also, significant (*p* = 0.004) differences were observed for marijuana-users compared with non-users for race/ethnicity with Whites reporting using most (32.7%), followed by Hispanics/Latino/a (27.6%) and then API (17.8%). Regarding grade point average (GPA), marijuana-users had a significantly (*p* = 0.009) lower GPA than non-users (*p* = 0.009), with a higher proportion of non-users reporting A’s (31.1%) compared with non-users (20.7%).

Upon examination of other high-risk behaviors (Table 3), marijuana-users consumed significantly more alcohol drinks during socializing compared with non-users (means: 3.88 ± 2.85 for users and 2.03 ± 2.62 for non-users, *p* < 0.0001). Furthermore, marijuana-users reported binge drinking (5 or more drinks in one setting) significantly more than non-users (2.80 ± 1.55; 1.84 ± 0.86, respectively; *p* < 0.0001). Also, marijuana-users were significantly (*p* < 0.0001) twice as likely to drive after drinking (25.1%) compared with non-users (12.1%) (*p* < 0.0001). Additionally, marijuana-users were five times (18.2%) more likely to smoke tobacco than non-users (2.9%), (*p* < 0.0001).

Sexual risk behaviors also yielded statistically significant differences among marijuana-users and non-users (*p* < 0.0001 for all behaviors). Marijuana-users reported 2.6 times as many sexual partners on average compared to non-users. Moreover, the reported use of a condom during vaginal intercourse was significantly (*p* < 0.0001) less likely among marijuana-users with 25.1% indicating never use, in relation to only 14.2% of non-users.

Multivariate logistic regression (Table 4) showed that after adjusting for demographic characteristics and high-risk behaviors, race/ethnicity was borderline significantly associated with marijuana use, specifically for Whites [OR = 1.53; 95% CI: (−0.01, 0.86), *p* = 0.06] and the Other race/ethnicity category [OR = 2.32; 95% CI: (0.12, 1.56), *p* = 0.02]. In addition, the number of drinks was significantly associated with marijuana use (OR: 1.16; 95% CI: (0.10–0.20); *p* < 0.0001), and so was the number of sexual partners (1.34; 95% CI: (0.20–0.38); *p* < 0.0001). Further, the odds ratio for tobacco users was 4.17 (95% CI: (0.89–1.96); *p* < 0.0001), indicating that tobacco users were 4.17 times more likely to be marijuana users compared to non-tobacco users.

## 6. Discussion

Our findings post legalization of marijuana for recreational use clearly demonstrated a higher prevalence of high-risk behaviors such as alcohol use, tobacco use, drinking and driving and sexual activities among college students who use marijuana compared to those who do not. Specifically, adjusting for all covariates in the logistic regression model, younger respondents were found to be more likely to be marijuana users; similarly, students earning D grades were 1.68 times more likely to use marijuana compared to those earning A grades (although not significant). Compared to APIs, Whites were 53% more likely and the Other race/ethnicity category were twice as likely to be marijuana users. These findings are consistent with similar studies examining risk-taking behaviors among marijuana-users for recreational use pre legalization [9-11,15-19,29,33-44].

This study is one of the first to assess marijuana use in an ethnically diverse student population. We found marijuana use was higher among White college students which mirrors most recent national data for 18-25-year-olds [4]. Interestingly, when examining marijuana-use disorders Pacek and colleagues [24] found African Americans twice as likely to be diagnosed with this condition versus both Whites and Hispanics. Due to small sample size of certain ethnic groups we did not specifically examine marijuana use among Blacks and Native American students. However, we found a strong relationship between the Other race/ethnicity category, (which predominantly comprised Blacks and Native Americans), and marijuana use, suggesting higher use of marijuana in this respective population. Nonetheless, further studies are needed with larger samples of African Americans and Native Americans.

While we found marijuana use slightly higher among males than females, it was not a statistically significant difference. National data shows men age 18 to 25 are more likely to use marijuana at least once a month compared to women [13,25,26]. However, when analyzing a 15-year trend, Johnson and colleagues [27] found that the male-female differences in marijuana use decreased over time; with the most recent SAMSA data indicating among 18-25 year-olds, males are only slightly higher in reported marijuana use than females [4].

In addition, we found an inverse relationship between academic performance and marijuana use; specifically, adjusting for all covariates in the logistic regression model, younger respondents were found to be more likely to use marijuana; similarly, students earning D grades were 1.68 times more likely to use marijuana compared to those earning A grades. This is similar to the findings from Arria and colleagues [8]. Their research found that not only did marijuana-users have lower grade point averages, but also skipped classes more and took longer to graduate college. Whether these negative outcomes are related to poor academic behaviors or poor cognitive function or both continues to be researched. In a review of the literature, Crane and colleagues [28] found that numerous studies continue to demonstrate the negative effect of cannabis on learning and memory, in addition to deficits in attention, concentration, and abstract reasoning.

With regard to alcohol use, our findings indicate that increased alcohol use is also associated with an increase in marijuana use. Recent studies exploring the role of marijuana policies on alcohol and marijuana use, show varying results. Some hypothesize that alcohol use may decrease as marijuana becomes legalized for recreation use as it would substitute for alcohol [29,30]. Others posit that with more liberal marijuana policies, both marijuana and alcohol use will increase as they may complement each other [31,32]. A critical review of the literature provides some evidence of both. The researchers of these studies note the issue is complex, suggesting likely factors such as how long the policy has been in place, how it is implemented as well as the age of users, play a role [33].

Tobacco was another substance we found marijuana smokers more likely to use. This was a significant finding as tobacco users were four times more likely to be marijuana users compared with non-tobacco users. Interestingly, some researchers have found that using nicotine with cannabis together can intensify the effects of cannabis [34-36]. In their study Ream and colleagues [37] found participants reported smoking cigarettes directly after using marijuana to enhance the intoxication. This may be due to the close overlapping in the distribution of brain receptors for nicotine and cannabis [38]. The relationship of smoking and marijuana use is supported by findings that show marijuana-users who also smoke cigarettes have increased risk of marijuana relapse when they are trying to quit [39].

As is the case with many substances, marijuana impairs judgement; therefore, we found a positive association between high-risk sexual behavior (# of sexual partners) and marijuana use [40,41]. Our univariate analyses also showed that marijuana-users in this study indicated they never use a condom during vaginal intercourse 25% of the time compared to only 14% of the time for non-users. These findings are similar to data showing marijuana use associated with non-use of condoms and having a higher number of sexual partners [16,42-44]. Some researchers assert this association may be due to marijuana use potentially increasing sexual desire or sensation [45,46] while others believe decreased cognition [47,48] and increased disinhibition [49] are contributing factors.

## 7. Strengths and limitations

Strengths and limitations of this study should be recognized. Although we assessed several behavioral factors among those who use marijuana and those who do not, we were limited by the number and types of items that addressed these behaviors. The National College Health Assessment is a comprehensive instrument designed to asses general student health status and engagement in a variety of health behaviors. In addition, because our population of college students was from one university, the findings are not generalizable to other age groups or college students outside of Southern California. With that said, it is worth noting the sample was large and was representative of the surrounding diverse metropolitan area. One other limitation includes the cross-sectional design which does not allow for causal inference regarding marijuana use and other health behaviors. Despite these limitations, this study is one of the first to provide insight into marijuana use post legalization for recreational purposes.

## 8. Implications and conclusions

Overall, this study provides data indicating high-risk health behaviors are more prevalent among college student marijuana-users than non-users post legalization. While propositions for the legalization of marijuana for recreational use expand across the United States, there is still much debate on the health and social impacts of marijuana use. Continued examination of marijuana use trends as well as health behavior trends is critical in monitoring the implications of policy changes. In addition, it will be important to be vigilant in the surveillance of vulnerable populations as we are seeing an increase in marijuana use among females and ethnic/racial groups. More specifically, college health educators and student affairs personnel should be aware of the results from studies like this and work to address marijuana use in college students and the other high-risk behaviors that likely accompany it.

## Figures and Tables

**Table 1:** Demographic and other characteristics of participants, (n = 1345).

Variable	
Age (mean ± SD).	23.10 ± 5.66
Gender, n (%).	
Male	343 (25.5%)
Female	1002 (74.5%)
All races	
White	353 (26.2%)
Hispanic/Latino/a	456 (33.9%)
Asian/Pacific Islander	288 (21.4%)
Multiracial/Biracial	195 (14.5%)
Other	53 (4.0%)
Approximate GPA.	
A	390 (29.0%)
B	745 (55.4%)
C	182 (13.5%)
D/F	7 (0.5%)
N/A	21 (1.6%)

**Table 2:** Demographic and other characteristics for marijuana users and non-users.

	Marijuana-users (n = 275)	Marijuana Non-users (n = 1070)	P-value
Age (mean ± SD)	22.27 ± 3.96	23.19 ± 6.00	0.016
Gender (n, %)			0.222
Male	78, 28.4%	265, 24.8%	
Female	197, 71.6%	805, 75.2%	
All races (n, %)			0.004
White	90, 32.7%	263, 24.6%	
Hispanic/Latino/a	76, 27.6%	380, 35.5%	
Asian/Pacific Islander	49, 17.8%	239, 22.3%	
Multiracial/Biracial	44, 16.0%	151, 14.1%	
Other	16, 5.8%	37, 3.5%	
Approximate GPA (n, %)			0.009
A	57, 20.7%	333, 31.1%	
B	164, 59.6%	581, 54.3%	
C	45, 16.4%	137, 12.8%	
D/F	2, 0.7%	5, 0.5%	
N/A	7, 2.5%	14, 1.3%	

**Table 3:** High-risk behaviors among marijuana users and non-users.

	Marijuana- users (n = 275)	Marijuana non-users (n = 1070)	p-value
Alcohol Consumption			
Number of drinks in last social setting (mean ± SD)	3.88 ± 2.85	2.03 ± 2.62	<0.0001
Five or more drinks of alcohol at a sitting (mean ± SD), last two weeks^a^	2.80 ± 1.55	1.84 ± 0.86	<0.0001
Driving after drinking any alcohol^b^ (n, %)			<0.0001
N/A, don’t drive	28, 10.2%	137, 12.8%	
N/A, don’t drink	13, 4.7%	325, 30.4%	
No	165, 60.0%	479, 44.8%	
Yes	69, 25.1%	129, 12.1%	
Driving after drinking five or more drinks of alcohol^b^ (n, %)			<0.0001
N/A, don’t drive	29, 10.5%	139, 13.0%	
N/A, don’t drink	15, 5.5%	325, 30.4%	
No	224, 81.5%	598, 55.9%	
Yes	7, 2.5%	8, 0.7%	
Tobacco [2] (n, %).			<0.0001
No	225, 81.8%	1,039, 97.1%	
Yes	50, 18.2%	31, 2.9%	
Sexual activity			
Number of sexual partners within the last 12 months, (mean ± SD)	2.33 ± 3.00	0.88 ± 1.37	<0.0001
Condom use, vaginal sex^b^ (n, %)			<0.0001
N/A, never did this sexual activity	24, 8.7%	410, 38.3%	
Have not done this sexual Activity^b^	49, 17.8%	167, 16.1%	
Never	69, 25.1%	152, 14.2%	
Rarely	28, 10.2%	58, 5.4%	
Sometimes	28, 10.2%	63, 5.9%	
Most of the time	32, 11.6%	74, 6.9%	
Always	45, 16.4%	146, 13.6%	

a Survey response options: 1 = N/A don’t drink, 2 = None, 3 = 1 time, 4 = 2 times, 5 = 3 times, 6 = 4 times, 7 = 5 times, 8 = 6 times (Note: times refers to # of times engaged in binge drinking).

b Activity/question time frame = within the last 30 days.

**Table 4:** The association [multivariable odds ratio (OR)] between demographic and high-risk behaviors with marijuana use.

	OR	95% CI	p-value
Age, yrs.	0.94	(−0.10, −0.03)	<0.001
Gender (ref = female)	0.89	(−0.46, 0.22)	0.48
Race/Ethnicity			
Asian/Pacific Islander (ref)	1.00	-	-
White	1.53	(−0.01, 0.86)	0.06
Hispanic/Latino/a	0.90	(−0.54, 0.33)	0.62
Multiracial/Biracial	1.13	(−0.38, 0.63)	0.63
Other	2.32	(0.12, 1.56)	0.02
Grade Point Average (GPA)			
GPA (ref = A)	1.00	-	-
GPA = B	1.26	(−0.12, 0.58)	0.19
GPA = C	1.27	(−0.25, 0.74)	0.34
GPA = D/F	1.68	(−1.20, 2.24)	0.56
Number of drinks last time/socialized	1.16	(0.10, 0.20)	<0.0001
Number of sexual partners	1.34	(0.20, 0.38)	<0.0001
Tobacco User	4.17	(0.89, 1.96)	<0.0001

## References

[1] “Cannabis overview’ national conference of state legislatures, https://www.ncsl.org/research/civil-and-criminal-justice/marijuana-overview.aspx.

[2] Volkow ND, Ruben MD, Baler D, Wilson M, Compton S, Weiss RB, Adverse health effects of marijuana use, N. Engl. J. Med 370 (23) (2014 4) 2219–2227. 24897085 10.1056/NEJMra1402309 PMC4827335

[3] “Support for Legal Marijuana Inches Up to New High of 68%.” Gallup, 11 9, 2020, https://news.gallup.com/poll/323582/support-legal-marijuana-inches-new-high.aspx.

[4] “National survey on drug use and health (NSDUH) presentation of the NSDUH data 2016-2019.” substance abuse and mental health service administration, https://www.samhsa.gov/data/release/2019-national-survey-drug-use-and-health-nsduh-releases, 2019.

[5] Schulenberg JE, Johnston LD, O’Malley PM, Bachman JG, Miech RA, Patrick ME, Monitoring the Future National Survey Results on Drug Use, 1975-2016: College Students and Adults Ages 19-55, Institute for Social Research, The University of Michigan, Ann Arbor, MI, 2017. Available at: http://monitoringthefuture.org/pubs/monographs/mtf-vol2_2016.pdf.

[6] McCabe SE, West BT, Wechsler H, Trends and college-level characteristics associated with the non-medical use of prescription drugs among U.S. college students from 1993 to 2001, Addiction 102 (3) (2007 3) 455–465. 17298654 10.1111/j.1360-0443.2006.01733.x PMC1838570

[7] Arria AM, Garnier-Dykstra LM, Caldeira KM, Vincent KB, Winick ER, O’Grady KE, Drug use patterns and continuous enrollment in college: results from a longitudinal study, J. Stud. Alcohol Drugs 74 (1) (2013 1) 71–83, 10.15288/jsad.2013.74.71. PMID: 23200152; PMCID: PMC3517265. 23200152 PMC3517265

[8] Arria AM, Caldeira KM, Bugbee BA, Vincent KB, O’Grady KE, The academic consequences of marijuana use during college, Psychol. Addict. Behav 29 (3) (2015 9) 564–575, 10.1037/adb0000108. 26237288 PMC4586361

[9] Meda SA, Gueorguieva RV, Pittman B, Rosen RR, Aslanzadeh F, Tennen H, Leen S, Hawkins K, Raskin S, Wood RM, Austad CS, Dager A, Fallahi C, Pearlson GD, Longitudinal influence of alcohol and marijuana use on academic performance in college students, PLoS One 12 (3) (2017 3) 1–16, 10.1371/journal.pone.0172213. PMC5342177 28273162

[10] Palfai TP, Tahaney KD, Winter MR, Is marijuana use associated with health promotion behaviors among college students? Health-promoting and health-risk behaviors among students identified through screening in a university student health services center, J. Drug Issues 46 (1) (2016 5) 41–50.

[11] Mohler-Kuo M, Lee JE, Wechsler H, Trends in marijuana use and other illicit drug use among college students: results from four Harvard School of Public Health College Alcohol Study Surveys: 1993–2001, J. Am. Coll. Health 52 (1) (2003 Jul-Aug) 17–24, 10.1080/07448480309595719. 14717576

[12] Johnston LD, O’Malley PM, & Bachman JG Monitoring the Future national results on adolescent drug use: Overview of key findings, 2002. 2004 NIH Publication No. 03–5374. Bethesda, MD: National Institute on Drug Abuse. www.monitoringthefuture.org/pubs/monographs/overview2004.pdf.

[13] “What are the effects of mixing marijuana with alcohol, tobacco or prescription drugs?” Centers for Disease Control and Prevention. https://www.cdc.gov/marijuana/faqs/mixing-marijuana-with-alcohol-tobacco-drugs.html, 3 7, 2018.

[14] A day in the life of college students aged 18 to 22: substance use facts NSDUH - national survey on drug use and health collected date: 2011-2014, in: Substance Abuse and Mental Health Service Administration, 2016. https://www.samhsa.gov/data/sites/default/files/NSDUH-DetTabs-2016/NSDUH-. 27854413

[15] Subbaraman MS, Kerr WC, Simultaneous versus concurrent use of alcohol and cannabis in the National Alcohol Survey, Alcohol Clin. Exp. Res 39 (5) (2015 5) 872–879. 25872596 10.1111/acer.12698 PMC4399000

[16] Berger AT, Khan MR, Hemberg JL, Race differences in longitudinal associations between personal and peer marijuana use and adulthood sexually transmitted infection risk, J. Addict. Dis 31 (2012) 130–142. 22540435 10.1080/10550887.2012.665691 PMC3340609

[17] Bryan AD, Schmiege SJ, Magnan RE, Marijuana use and risky sexual behavior among high-risk adolescents: trajectories, risk factors, and event-level relationships, Dev. Psychol 48 (5) (2012) 429–442, 10.1037/a0027547. 22390666 PMC12239880

[18] Guo J, Chung IJ, Hill KG, Hawkins JD, Catalano RF, Abbott RD, Developmental relationships between adolescent substance use and risky sexual behavior in young adulthood, J. Adolesc. Health 4 (2002) 354–362. 10.1016/s1054-139x(02)00402-0 12359381

[19] Metrik J, Caswell AJ, Magill M, Monti PM, Kahler CW, Sexual risk behavior and heavy drinking among weekly marijuana users, J. Stud. Alcohol Drugs 77 (1) (2016) 104–112. 26751360 10.15288/jsad.2016.77.104 PMC4711311

[20] Keyes KM, Wall M, Feng T, Cerdá M, Hasin DS, Race/ethnicity and marijuana use in the United States: diminishing differences in the prevalence of use, 2006–2015, Drug Alcohol Depend. 179 (2017) 379–386. 28846954 10.1016/j.drugalcdep.2017.07.027 PMC5599376

[21] Bae H, Kerr DCR, Marijuana use trends among college students in states with and without legalization of recreational use: initial and longer-term changes from 2008 to 2018, Addiction 115 (6) (2020 6) 1115–1124, 10.1111/add.14939. Epub 2020 Jan 13. PMID: 31833119. 31833119

[22] Alley ZM, Kerr DCR, Bae H, Trends in college students’ alcohol, nicotine, prescription opioid and other drug use after recreational marijuana legalization: 2008-2018, Addict. Behav 102 (2020 3) 106212, 10.1016/j.addbeh.2019.106212. Epub 2019 Nov 11. PMID: 31846837. 31846837

[23] Kerr DCR, Bae H, Phibbs S, Kern AC, Changes in undergraduates’ marijuana, heavy alcohol and cigarette use following legalization of recreational marijuana use in Oregon, Addiction 112 (11) (2017 11) 1992–2001, 10.1111/add.13906. Epub 2017 Jul 11. PMID: 28613454. 28613454

[24] Pacek LR, Malcolm RJ, Martins SS, Race/ethnicity differences between alcohol, marijuana, and co-occurring alcohol and marijuana use disorders and their association with public health and social problems using a national sample, Am. J. Addict 21 (5) (2012 9) 435–445. 22882394 10.1111/j.1521-0391.2012.00249.x PMC3419382

[25] Carliner H, Mauro PM, Brown QL, Shmulewitz D, Rahim-Juwel R, Sarvet AL, Wall MM, Martins SS, Carliner G, Hasin DS, The widening gender gap in marijuana use in the U.S. during a period of economic change, 2002-2014, J. Alcohol. Drug 170 (2017 1) 51–58. 10.1016/j.drugalcdep.2016.10.042 PMC5321672 27875801

[26] Hasin DSUS, Epidemiology of cannabis use and associated problems, Neuropsychopharmacology 43 (1) (2018 8) 195–212, 10.1038/npp.2017.198. 28853439 PMC5719106

[27] Johnson RM, Fairman B, Gilreath T, Xuan Z, Rothman EF, Parnham T, Furr-Holden CD, Past 15-year trends in adolescent marijuana use: differences by race/ethnicity and sex, J. Alcohol. Drug 155 (10 2015) 8–15. 10.1016/j.drugalcdep.2015.08.025 PMC4582007 26361714

[28] Crane NA, Schuster RM, Fusar-Poli P, Gonzalez R, Effects of cannabis on neurocognitive functioning: recent advances, neurodevelopmental influences, and sex differences, Neuropsychol. Rev 23 (2) (2013) 117–137. 23129391 10.1007/s11065-012-9222-1 PMC3593817

[29] Anderson DM, Hansen B, Rees DI, Medical marijuana laws, traffic fatalities, and alcohol consumption, J. Law Econ 56 (2) (2013) 333–369.

[30] Kilmer B, Recreational cannabis - minimizing the health risks from legalization, N. Engl. J. Med 376 (8) (2017 2) 705–707. 28225673 10.1056/NEJMp1614783

[31] Hall W, Lynskey M, Evaluating the public health impacts of legalizing recreational cannabis use in the United States, Addiction 111 (10) (2016 10) 1764–1773. 27082374 10.1111/add.13428

[32] Hawkins EJ, Malte CA, Grossbard JR, Saxon AJ, Prevalence and trends of concurrent opioid analgesic and benzodiazepine use among Veterans Affairs patients with post-traumatic stress disorder, 2003-2011, Pain Med. 16 (10) (2015) 1943–1954. 26332513 10.1111/pme.12787

[33] Guttmannova K, Lee CM, Kilmer JR, Fleming CB, Rhew IC, Kosterman R, Larimer ME, Impacts of changing marijuana policies on alcohol use in the United States, Alcohol Clin. Exp. Res 40 (1) (2015 12) 33–46. 26727520 10.1111/acer.12942 PMC4700545

[34] Penetar DM, Kouri EM, Gross MM, McCarthy EM, Rhee CK, Peters EN, Transdermal nicotine alters some of marihuana’s effects in male and female volunteers, Alcohol Drug Depend 79 (2005) 211–223. 10.1016/j.drugalcdep.2005.01.008 16002030

[35] Rabin RA, George TP, A review of co-morbid tobacco and cannabis use disorders: possible mechanisms to explain high rates of co-use, Am. J. Addict 24 (2) (2015) 105–116. 25662704 10.1111/ajad.12186

[36] Wang JB, Ramo DE, Lisha NE, Cataldo JK, Medical marijuana legalization and cigarette and marijuana co-use in adolescents and adults, Drug Alcohol Depend. 166 (2016 9) 32–38, 10.1016/j.drugalcdep.2016.06.016. 27460859 PMC4983542

[37] Ream GL, Benoit E, Johnson BD, Dunlap E, Smoking tobacco along with marijuana increases symptoms of cannabis dependence, Drug Alcohol Depend. 95 (3) (2008 6) 199–208. 18339491 10.1016/j.drugalcdep.2008.01.011 PMC2440496

[38] Viveros MP, Marco EM, File SE, Nicotine and cannabinoids: parallels, contrasts and interactions, Neurosci. Biobehav. Rev 30 (2006) 1161–1181. 17049986 10.1016/j.neubiorev.2006.08.002

[39] Haney M, Gillinder B, Ziva D, Cooper A, Glass SK, Vosburg SD, Comer RW, Foltin R, Predictors of marijuana relapse in the human laboratory: robust impact of tobacco cigarette smoking status, Biol. Psychiatr 73 (3) (2013) 242–248, 10.1016/j.biopsych.2012.07.028. PMC3522776 22939992

[40] Lane SD, Cherek DR, Tcheremissine OV, Lieving LM, Pietras CJ, Acute marijuana effects on human risk taking, Neuropsychopharmacology 30 (2005 3) 800–809. 15775958 10.1038/sj.npp.1300620

[41] Poulin C, Graham L, The association between substance use, unplanned sexual intercourse and other sexual behaviors among adolescent students, Addiction 96 (4) (2001 4) 607–621. 11300964 10.1046/j.1360-0443.2001.9646079.x

[42] Anderson BJ, Stein MD, A behavioral decision model testing the association of marijuana use and sexual risk in young adult women, AIDS Behav. 15 (4) (2011 5) 875–884, 10.1007/s10461-010-9694-z. 20358274 PMC2916938

[43] Beilis MA, Hughes K, Calafat A, Juan M, Ramon A, Rodriguez JA, Phillips-Howard P, Sexual uses of alcohol and drugs and the associated health risks: a cross sectional study of young people in nine European cities, BMC Publ. Health 8 (2008 5) 155–165, 10.1186/1471-2458-8-155. PMC2409330 18471281

[44] Patrick ME, O’Malley PM, Johnston LD, HIV/AIDS risk behaviors and substance use by young adults in the United States, Prev. Sci 13 (5) (2012 10) 532–538, 10.1007/s11121-012-0279-0. 22886042 PMC3586255

[45] Gorzalka BB, Hill MN, Chang SC, Male–female differences in the effects of cannabinoidson sexual behavior and gonadal hormone function, Horm. Behav 58 (1) (2010) 91–99, 10.1016/j.yhbeh.2009.08.009. 19733173

[46] Palamar JJ, Acosta P, Ompad DC, Friedman SR, A qualitative investigation comparing psychosocial and physical sexual experiences related to alcohol and marijuana use among adults, Arch. Sex. Behav 47 (3) (2018) 757–770. 27439599 10.1007/s10508-016-0782-7 PMC5250581

[47] Harvey MA, Sellman JD, Porter RJ, Frampton CM, The relationship between non-acute adolescent cannabis use and cognition, Drug Alcohol Rev. 26 (3) (2007) 309–319, 10.1080/09595230701247772. 17454021

[48] Thoma RJ, Monnig MA, Lysne PA, Ruhl DA, Pommy JA, Bogenschutz M, Yeo RA, Adolescent substance abuse: the effects of alcohol and marijuana on neuropsychological performance, Alcohol Clin. Exp. Res 35 (1) (2011) 1–8. 20958330 10.1111/j.1530-0277.2010.01320.x PMC3005001

[49] Skosnik PD, Spatz-Glenn L, Park S, Cannabis use is associated with schizotypy and attentional disinhibition, J. Schizophr. Res. 48 (1) (2001 3) 83–92, 10.1016/s0920-9964(00)00132-8. 11278156

